# Recurrent painful blisters over the right foot

**DOI:** 10.1016/j.jdcr.2024.10.010

**Published:** 2024-10-30

**Authors:** An Jian Leung, Zhao Jian Oswald Lee, Lester Juay

**Affiliations:** a Department of Dermatology, National University Hospital, Singapore, Singapore; b Department of Pathology, National University Hospital, Singapore, Singapore

**Keywords:** blisters, genetics, immunoblistering diseases, livedoid vasculopathy

## Case description

**Fig 1:**
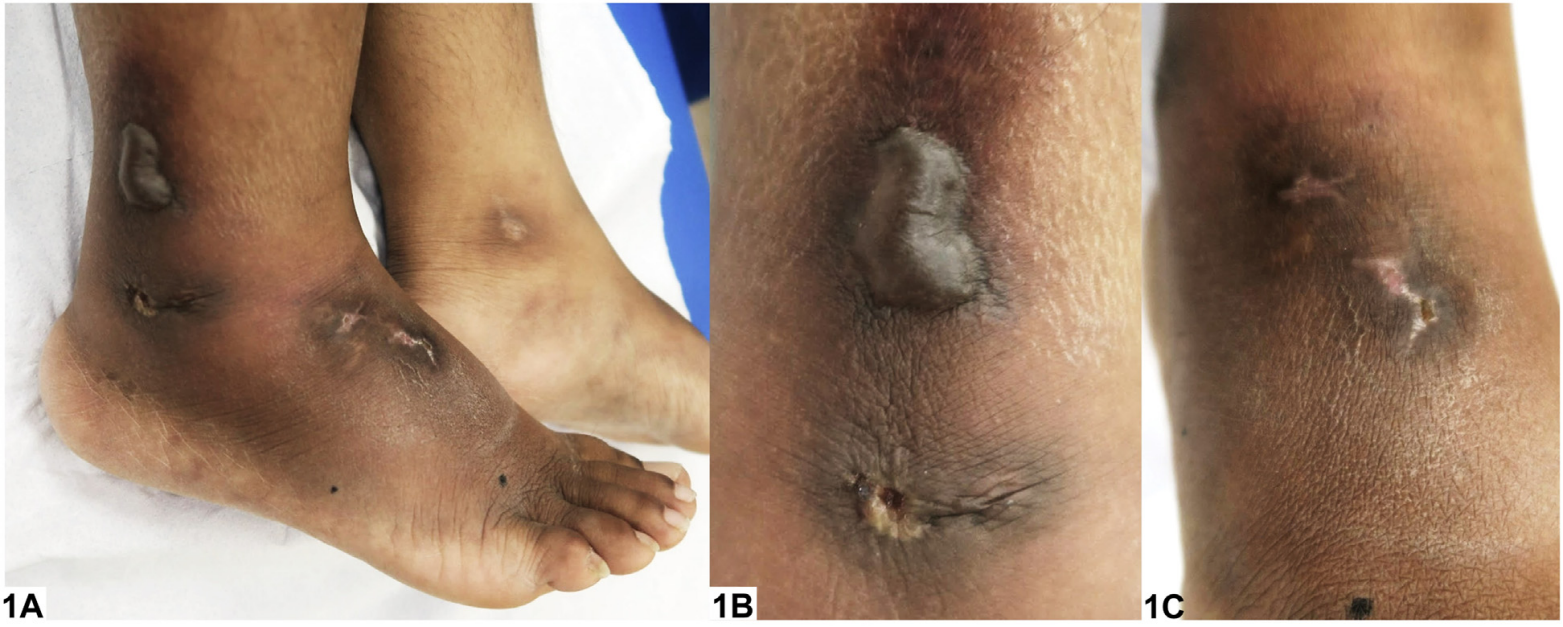

**Fig 2:**
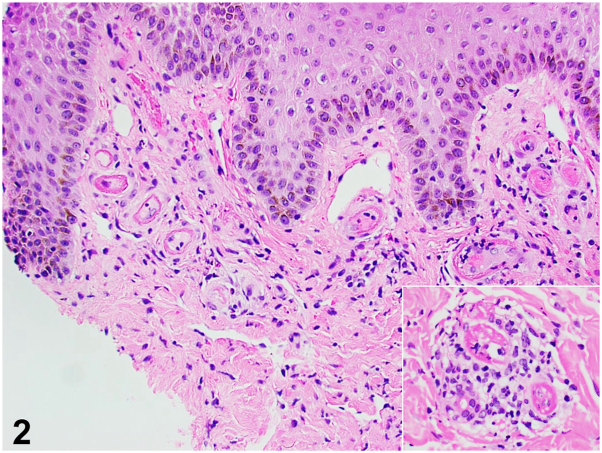

A previously well 18-year-old female of Indian descent presented with a 2-year history of recurrent right foot blisters after minor trauma. The painful tense blisters were confined to the distal right lower limb. She had received multiple courses of antibiotics to poor effect. On examination, a single tense bulla with hyperpigmented rim was seen on the right lateral malleolus (Fig 1, *A* and *B*). Previous blister sites healed as stellate atrophic scars with peripheral hyperpigmentation (Fig 1, *A-C*). A punch biopsy was performed, and histological findings are shown below (Fig 2). Direct immunofluorescence revealed heavy fibrin exudates within the blood vessels.

**Question 1: What is the most likely diagnosis?**

A. Bullous contact dermatitis
B. Bullous leukocytoclastic vasculitis
C. Bullous livedoid vasculopathy (LV)
D. Bullous pemphigoid
E. Bullous tinea pedis

**Answers:**

A. Bullous contact dermatitis – Incorrect. The lack of a contactant history and the absence of epidermal spongiosis with dermal eosinophilia on histology make this an unlikely diagnosis.
B. Bullous leukocytoclastic vasculitis – Incorrect. While leukocytoclastic vasculitis may present as recurrent bullous papules and plaques on the lower limb, the clinical absence of purpuric tender papules and histologic absence of leukocytoclasia and fibrinoid necrosis of vessel walls render this diagnosis less likely.
C. Bullous livedoid vasculopathy (LV) – Correct. Bullous LV is a rare thrombotic dermal vasculopathy that presents with recurrent painful blisters typically over the lower limb. Healing with stellate atrophie blanche is characteristic.^1^^,^^2^ Surrounding erythema expected in LV was less evident here given this patient’s darker skin. Instead, hyperpigmentation was observed. Hyalinized vessels with microthrombi on histology confirm the diagnosis.^2^ Treatment involves smoking cessation, debridement of slough, and antibiotics for superimposed cellulitis. When clotting disorders are identified, antiplatelets, anticoagulants, or fibrinolytics may be used.
D. Bullous pemphigoid – Incorrect. Bullous pemphigoid is a subepidermal blistering disorder characterized by generalized itchy tense fluid-filled blisters. Furthermore, the presence of microthrombi, tufts of small vessels, and hyalinized walls with lack of subepidermal split on histology makes this diagnosis less likely.
E. Bullous tinea pedis – Incorrect. Bullous tinea pedis may occur in dermatophyte infections, though the absence of annular scaly plaques and hyphae on histology, as well as the presence of stellate scarring, makes this diagnosis unlikely.

**Question 2: Which of the following are causes of LV?**

A. Antiphospholipid syndrome (APS)
B. Hemophilia A
C. Hepatitis B
D. Sjogren’s syndrome
E. Ulcerative colitis

**Answers:**

A. Antiphospholipid syndrome (APS) – Correct. APS is characterized by the presence of APS antibodies and recurrent thrombosis. Apart from LV, APS can cause livedo reticularis, livedo racemosa, malignant atrophic papulosis, or cutaneous ulcers.^3^ Prothrombotic states like factor V leiden, protein C and S deficiency, or homocysteinemia are also established causes for LV.^3^
B. Hemophilia A – Incorrect. Hemophilia A is a genetic clotting disorder characterized by factor VIII deficiency. It causes easy bleeding, not thrombosis.
C. Hepatitis B – Incorrect. Cutaneous manifestations of hepatitis B include polyarteritis nodosa, cryoglobulinemia, and antineutrophil cytoplasmic antibody (ANCA)-positive vasculitis.
D. Sjogren’s syndrome – Incorrect. While secondary APS may be associated with Sjogren’s syndrome, LV is unassociated with Sjogren’s syndrome in isolation.
E. Ulcerative colitis – Incorrect. Inflammatory bowel disease can manifest cutaneously with pyoderma gangrenosum, erythema nodosum, and rarely, Sweet’s syndrome. LV is not associated with inflammatory bowel disease.

**Question 3: What is the genetic mutation most commonly associated with LV?**

A. Dystonin gene
B. FERM domain-containing kindlin 1
C. Human leukocyte antigen DR beta (HLA-DRB) 1
D. Keratin 5
E. Prothrombin G20210A

**Answers:**

A. Dystonin gene – Incorrect. This mutation is associated with epidermolysis bullosa simplex and hereditary sensory and autonomic neuropathy type VI.
B. FERM domain-containing kindlin 1 – Incorrect. This mutation is associated with epidermolysis bullosa.
C. HLA-DRB1 – Incorrect. This mutation is associated with pemphigus vulgaris.
D. Keratin 5 – Incorrect. This mutation is associated with epidermolysis bullosa simplex.
E. Prothrombin G20210A – Correct. Eight percent of patients with LV carry a mutation in this gene.^4^

## Conflicts of interest

None disclosed.
